# Study of Androgenic Plant Families of Alloplasmic Introgression Lines (*H. vulgare*) –*T. aestivum* and the Use of Sister DH Lines in Breeding

**DOI:** 10.3390/plants9060764

**Published:** 2020-06-18

**Authors:** Lidiya Pershina, Nataliya Trubacheeva, Ekaterina Badaeva, Igor Belan, Ludmila Rosseeva

**Affiliations:** 1 Institute of Cytology and Genetics, SB RAS, 630090 Novosibirsk, Russia; pershina@bionet.nsc.ru; 2 Vavilov Institute of General Genetics RAS, 119991 Moscow, Russia; iogen@vigg.ru; 3 Omsk Agricultural Scientific Center, 644012 Omsk, Russia; belan_skg@mail.ru (I.B.); rosseeva@mail.ru (L.R.)

**Keywords:** anther culture, families of regenerants, (*H. vulgare*)–*T. aestivum* lines, breeding

## Abstract

One of the limitations in obtaining the genetic diversity of doubled haploid (DH) lines via anther culture is the development of families of regenerants, and each family represents a clone. This work examines the results of studying this phenomenon in anther culture of alloplasmic (*H. vulgare*)–*T. aestivum* and euplasmic lines with 1RS.1BL and 7DL-7Ai translocations and hybrids between them. Parameters of androgenesis such as the number of embryo-like structures, the total number of regenerants, and the number of green regenerants per 100 anthers varied depending on the genotype. In all genotypes from embryo-like structures, predominant development of families of plantlets rather than single plantlets was found. The source of family plantlets was polyembryos. About 75% of families consisted of regenerants at the same fertility level. On average, 37.74%4% of the R0 plants were fertile. The sister DH lines of three hybrid combinations were formed from seeds of R1 plants (2*n* = 42) with high fertility and in the presence of wheat–alien translocations. After four years of breeding trials, the sister DH lines of three families with fungal disease resistance increased yield, and some parameters of grain quality exceeding the controls were identified as promising for breeding.

## 1. Introduction

One of the goals of increasing the genetic diversity of bread wheat is to create new genotypes that are resistant to fungal pathogens that affect plants, leading to decreased grain yield and quality [1]. The main approach to obtaining new bread wheat genotypes is introgressive hybridization aimed at transferring valuable genes from cultivated and wild relatives to their genome [2,3]. Alloplasmic lines combining the nuclear genome of one species with the cytoplasm of another species are considered to be an additional source of biodiversity for cultivated plants [4]. The new nuclear–cytoplasmic interactions in alloplasmic genotypes can cause epigenetic modifications of nuclear genes [5], leading to changes at the level of transcription and metabolism [5,6]. Given that variability in the chloroplast and mitochondrial genomes contributes significantly to the adaptation of plants to biotic and abiotic environmental factors [7], alloplasmic lines with restored fertility can be valuable genotypes for creating new varieties of bread wheat [8], including those with introgression of alien genes [9].

Earlier, we reported the production of recombinant alloplasmic lines of bread wheat derived from backcross progenies of barley–wheat hybrid *H. vulgare* × *T. aestivum* and restoration of their fertility [10,11]. Alloplasmic (*H. vulgare*)–*T. aestivum* lines characterized by different fertility levels proved to be valuable models for studying the variability of both nuclear and organellar (mitochondrial and chloroplast) genomes in the process of nuclear–cytoplasmic co-adaptation as a result of backcrossing wide hybrids with paternal genotypes [12,13,14]. Some of the recombinant alloplasmic (*H. vulgare*)–*T. aestivum* lines with full fertility restoration and their doubled haploid (DH) lines were effectively used to obtain new introgression genotypes [14,15] and their involvement in breeding [16]. As a result of selections in different regions, alloplasmic lines L-311(4), L-311(5), and L-311(6), carrying translocation 1RS.1BL with gene complex *Lr26*/*Sr3*1/*Yr9*/*Pm8* controlling resistance to fungal pathogens, became commercially valuable varieties of spring bread wheat (Uralosibirskaya 2, Sigma, and Ishimskaya 11, respectively) [15,16]. Varieties with the 1RS.1BL translocation are widespread [17]. Mass cultivation of such varieties has resulted in the appearance of a highly aggressive race of stem rust, Ug99, virulent to the gene *Sr31* [18]. Currently, the gene *Sr31* in Russia remains effective in protecting against stem rust [19], but the genes *Yr9* [20] and *Pm8* [21] linked to *Sr31* have lost their effectiveness, and the gene *Lr26* is most effective in combination with other leaf rust resistance genes [22]. In order to ensure the protection of wheat varieties from the possible spread of the race Ug99 as well as other fungal pathogens, it is necessary to conduct pyramiding of disease resistance genes in the genetic background of highly productive genotypes. Alloplasmic (*H. vulgare*)–*T. aestivum* lines of the L-311 group were used as such genotypes in our research. Resistance gene donors were lines derived from wheat varieties Omskaya 37 and Omskaya 38 with translocations 1RS.1BL and 7DL-7Ai (carrier of genes *Lr19*/*Sr25*) and showed resistance to stem rust when studied in an international nursery in Kenya [23], as well as lines with introgression of genes from different species of *Triticum*. 

Developing a new variety is a lengthy and costly process; therefore, this process is accelerated by using homozygous DH lines in many breeding programs [24,25]. DH lines are created within one year and can be quickly analyzed in repeated trials, accelerating the selection of genotypes with the desired traits in the process of variety development [26], especially in combination with marker-assisted selection (MAS) [27]. When using traditional methods to stabilize desired traits, for example, in bread wheat, it is necessary to obtain six to eight self-pollinated generations [24]. DH lines are successfully used in hybridization to create new breeding material [28] since their use makes it possible to fix in one genotype a combination of a series of target genes (gene pyramiding) introgressed from different parents. This can provide long-term resistance to biotic stress [29], obtain genotypes with resistance to abiotic factors [30], or lead to fixation of heterosis [31]. Obtaining DH lines is an important and widely used method for the selection of mutations [32], for the detailed study of many traits in plants, including quantitative ones [33,34], transformation [35]; and genomic editing [36].

Several technologies are used to produce DH wheat lines. One includes the crossing of wheat with haploproducers (e.g., *Zea mays* and *Imperata cylindrica*) [37]. In such hybrid combinations, haploproducer chromosomes are eliminated at the early stages of embryo development, which leads to the development of haploid wheat embryos. Using embryo rescue techniques makes it possible to grow haploid plants, which, after doubling the number of chromosomes, become sources of DH lines. Another approach for producing DH lines is based on the induction of androgenesis in anther [38,39] and isolated microspore culture [39]. Stressful conditions are created that cause the reprogramming of microspores from the gametophyte to the sporophyte pathway during in vitro cultivation [40]. This leads to the formation of embryo-like structures (androgenic structures) from microspores on the induction culture medium. Embryo-like structures develop into seedlings during cultivation on regeneration medium. The efficiency of anther and microspore culture methods is measured by the frequency of obtaining viable green plants in order to form DH lines that are necessary for further work [41]. 

Potentially, under culture conditions, each microspore can become a source of one androgenic plant, and one anther, for example, of bread wheat, contains more than 1500 microspores [42]. However, many factors limit the production of androgenic plants in anther culture. The success of androgenesis induction in anther culture is influenced by the conditions of the growth of donor plants; the methods of anther pretreatment; the stage of development of microspores; the composition of culture medium, in which there is an important role for growth regulators; and the culture conditions of anthers and androgenic structures [26]. Despite the possibility of optimizing the whole complex of methods, the reaction of anthers to the culture conditions is determined by the influence of the plant genotype [43,44]. This is due to the fact that each main stage of androgenesis (embryo regeneration, embryoid induction to seedling regeneration, development of green seedlings and albinos) is under independent genetic control by the nuclear genome [45] and cytoplasm [46]. Restrictions in the production of DH lines by anther and microspore cultures are associated with gametoclonal variation, particularly for plants that have regenerated from gametic cells under in vitro conditions. Typical manifestations of gametoclonal variation associated with deletions in the chloroplast genome are the development of albinism [47] and changes in the number of chromosomes and their structure [41,48]. However, spontaneous doubling of the number of chromosomes in regenerants leads to the restoration of fertility, which excludes their treatment with colchicine [48]. Cytogenetic variation in androgenic plants is especially pronounced when hybrid genotypes characterized by cytogenetic instability in meiosis are used as donors for anther and microspore cultures [41,48,49]. Another serious limitation of the use of DH lines in breeding and genetic research is reduced genetic diversity through the formation of genetically identical plants, i.e., clones, among androgenic regenerants [50]. These authors emphasize that this problem is practically ignored in research papers. 

Earlier in our work, we noted that in the anther cultures of some wheat genotypes, clusters (families) of regenerants developed along with single regenerants [48]. However, it was unclear whether the formation of regenerant families occurs only in certain genotypes of bread wheat or is typical for many of them. In addition, when transferring embryoid-like structures (ELS) to the regeneration medium, it was not controlled how far they were separated from each other. The aim of this work was to study the parameters of androgenesis and the formation of regenerant families in the anther culture of introgression alloplasmic wheat genotypes (*H. vulgare*)–*T. aestivum* when creating promising DH lines for breeding carriers of a complex of genes for resistance to fungal pathogens. In this regard, the objectives of the study were:

- to determine how often the formation of regenerant families occurs in comparison with the development of single plantlets in the anther culture of bread wheat genotypes of different origin;
- to characterize families of regenerants R0 by the level of fertility, which was manifested in plants as a result of spontaneous doubling of the number of chromosomes;
- to study the association of reduced fertility in R1 regenerants with aneuploidy;
- to evaluate the prospects of alloplasmic sister DH lines for further breeding based on field trials studying resistance to fungal pathogens and agronomic traits.

## 2. Results

### 2.1. Development of Embryo-Like Structures and Plant Regeneration

**Figure 1:**
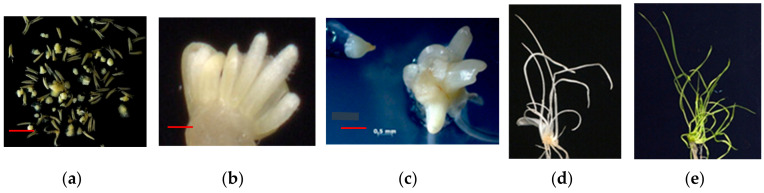
(**a**) Embryo-like structures (ELS) developed in anther culture after four weeks of cultivation (bar = 5 mm). (**b**,**c**) Polyembryos developed from ELS (b: bar = 2 mm; c: bar = 1.5 mm); (**d**) cluster of albinos (bar = 10 mm) and (**e**) green plantlets (bar = 15 mm) developed from polyembryos.

The development of embryo-like structures (ELS) was induced in the anther culture of each tested genotype. The appearance of ELS was observed in 3–4 weeks from the beginning of cultivation (Figure 1a).

The average number of ELS/100 anthers was 56.08, and the value ranged from 8.93 to 124.46 ELS/100 anthers depending on genotype (Table 1). The lowest value was in the Om38 euplasmic line, and the highest value was in the L-311(4) alloplasmic line. The group with low ELS values except Om38 includes the euplasmic Om37 lines and alloplasmic L-17(3). The values of the four hybrid combinations (L-311(4) × Om37, Om37 × L-311(4), L-311(4) × L-134, and L-311(4) × Om38) did not differ from each other. ELS values in reciprocal hybrids L-311(4) × Om37 and Om37 × L-311(4) were average compared to ELS values of parent genotypes. Approximately seven days after the start of ELS cultivation, single plantlets and clusters (families) of plantlets began to develop in a regeneration medium environment. Sources of families of plantlets were polyembryos (Figure 1b,c). From one polyembryo, either only albinos (Figure 1d) or only green plantlets (GPs) (Figure 1e) developed. 

The total number of all plantlets (albino and GP) per 100 anthers was estimated, including single albinos and GPs and regenerants from all families, and the average was 44.04/100 anthers. The number of all regenerants ranged from 3.16 to 105.2 per 100 anthers depending on genotype (Table 1). The minimum value was in the Om38 line, and the highest value was in the L-311(4) line. In reciprocal hybrids L-311(4) × Om37 and Om37 × L-311(4), as in the hybrid combination L-311(4) × Om38, the number of all regenerants/100 anthers was higher than in the euplasmic parent line (Om37 and Om38) but lower than in the alloplasmic line L-311(4). In hybrid combinations L-311(4) × L-134 and L-311(4) × 2870, the number of all regenerants/100 anthers was also lower than in the alloplasmic L-311(4) line. In some genotypes, the number of all plantlets/100 anthers exceeded the number of ELS/100 anthers. In the L-17(3) line, the number of ELS/100 anthers was 10.76, and the number of all plantlets/100 anthers was 16.60; in the hybrid combination L-311(4) × Om37, these values were 65.51 and 78.03, respectively, and in the combination L-311(4) × 2870, these values were 46.05 and 51.23, respectively (Table 1). 

The average number of GPs/100 anthers was 21.99 and ranged from 0.20 to 61.76, depending on genotype. The smallest number of GPs/100 anthers was in the euplasmic Om38 line, and the highest was in the alloplasmic L-311(4) line. In reciprocal hybrids L-311(4) × Om37 and Om37 × L-311(4), the number of GPs/100 anthers was higher than in euplasmic parent line Om37 but lower than in alloplasmic parent line L-311(4). In the other hybrid combinations L-311(4) × L-134, L-311(4) × Om38, and L-311(4) × 2870, the number of GPs/100 anthers was also lower than in alloplasmic parent line L-311(4). 

### 2.2. Efficiency of Development of Single Green Plantlets and Green Plantlet Families from ELS 

The efficiency of the development of single GPs and their families as a result of the cultivation of ELS was studied. The number of single GPs ranged from 0 in the Om38 line to 1.91/100 ELS in the hybrid combination L-311(4) × Om38 (Table 2). The number of GP families ranged from 2.27/100 ELS in the Om38 line to 8.42/100 ELS in the hybrid Om37 × L-311(4). On average, the number of GP families/100 ELS was significantly higher for all genotypes in comparison with the number of single GPs/100 ELS. Only the Om38 line had no significant differences between the number of single GPs and GP families/100 ELS. On average, 5.80 GPs developed in one family, ranging from 2 (Om38 line) to 36 (hybrid L-311(4) × Om37). The number of GPs in one family ranged from 2–10 in Pyr28 to 2–36 in L-311(4) × Om37. 

### 2.3. Evaluation of Families and Androgenic R0 plants

**Figure 2:**
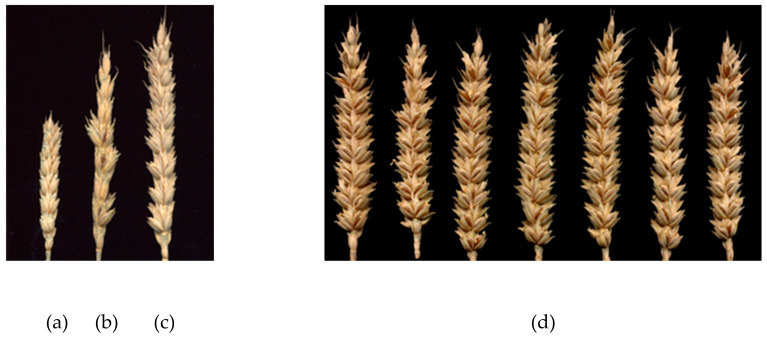
Spikes of R0 plants: (**a**) sterile, (**b**) partly fertile; (**c**) fertile. Spikes of R0 sister plants from one family of hybrid combination L-311(4) × 2870 (**d**).

A total of 220 families of green plants that reached heading were analyzed. Sterile (S), partially fertile (PF), and fertile (F) plants were identified among the androgenic plants. Figure 2a,b,c shows the spikes of androgenic R0 plants with different levels of fertility.

Four main types of families of sister green plants were identified in all tested genotypes (Table 3). Families consisting of S sister plants were found with the highest frequency (46.82%). The average frequency of families with PF sister plants was 9.09%, ranging from 5.56% for the hybrid L-311(4) × L-134 to 15.38% for the L-17(3) line. The frequency of families with F sister plants averaged 20.00%, ranging from 5.56% for the hybrid L-311(4) × Om38 to 38.30% for the hybrid combination L-311(4) × 2870. Figure 2d shows the spikes of fertile sister plants of one family of this hybrid. On average, 24.09% of the families consisted of separate clusters of families that differed from one another in the fertility of their sister plants. In some cases, the clusters were separated from each other during harvesting, after clay pellets were removed from the root. In the tested families, among 1314 androgenic R0 plants that reached heading, 496 plants (37.74%) set seeds (Table 4). The minimum number of such plants was in the hybrid L-311(4) × Om38 (18.88%), and the maximum was in the hybrid L-311(4) × L-2870 (52.73%). 

### 2.4. Evaluation of Sister Line Families in R1 

The results of studying the level of fertility, estimated by the number of seeds set in the main spike, in 52 families of R1 sister lines from four alloplasmic genotypes are presented in Table 5. Three groups of families were identified by the level of fertility in plants of sister lines. In group I, in 50.00% of families on average, all R1 sister lines consisted only of fertile plants with more than 15 grains in the main spike. The frequency of such families ranged from 36.84% in the hybrid combination L-311(4) × 2870 to 80.00% in the L-311(4) line. In group II, the frequency of fertile plants in sister lines ranged from 75% to 95%, and single plants were sterile or partially fertile. The frequency of such families ranged from 13.33% for the L-311(4) line to 47.37% for the hybrid combination L-311(4) × 2870. Group III is a family with an evident segregation of sister lines into fertile, partially fertile, and sterile plants. In group III, the frequency of fertile plants in sister lines was less than 50%. The frequency of families in group III ranged from 6.67% in the L-311(4) line to 37.50% in the hybrid combination L-311(4) × L-134. 

A comparison was made between the studied indicators in each group of families in the L-311(4) line compared to the average of the three hybrid combinations. In the L-311(4) line, the frequency of families with only fertile plants in the sister lines (group I) was 80.00%, which is significantly higher than the average of the three hybrid combinations (37.84%) (Table 5). In contrast, in groups II and III of families in which the plants of sister lines were segregated into fertile, partially fertile, and sterile plants, the frequency of the L-311(4) line families was significantly lower than the average for the three hybrid combinations. Significant differences were also found in the frequency of families in groups I and II between the L-311(4) line and the hybrid combination L-311(4) × 2870. 

The results of cytogenetic analysis of plants from sister lines of R1 families of four genotypes are summarized in Table 6. Fertile plants from groups I and II and partially fertile plants from groups II and III were analyzed. It was found that not all fertile plants were hexaploid. On average, aneuploids were detected among fertile plants with a frequency of 9.86%. 

The variation of the frequency of aneuploids was from 5.13% in L-311(4) to 14.58% in the hybrid combination L-311(4) × 2870 among fertile plants. Some genotypes also had hexaploids and aneuploids among partially fertile plants, and on average the frequency of aneuploids (86.00%) was significantly higher than that of hexaploids (14.00%). Variation in the frequency of aneuploids in partially fertile lines was from 73.33% in the hybrid combination L-311(4) × 2870 to 100% in L-311(4) × Om37. 

**Figure 3:**
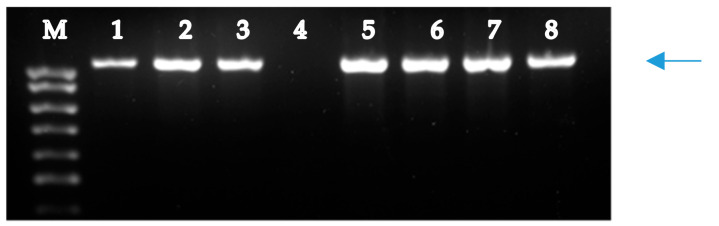
PCR amplification pattern of the marker iag95 linked to gene *Lr26*. Lanes: 1, rye *S. cereale* (control), 2, L-311(4); 3, Om37; 4, Pyr 28; 5, L-311(4) × Om37; 6, L-311(4) × L-134; 7-8, L-311(4) × 2870; M, 100 bp DNA ladder. Arrow indicates the 1100-bp band specific for *S. cereale*.

**Figure 4:**
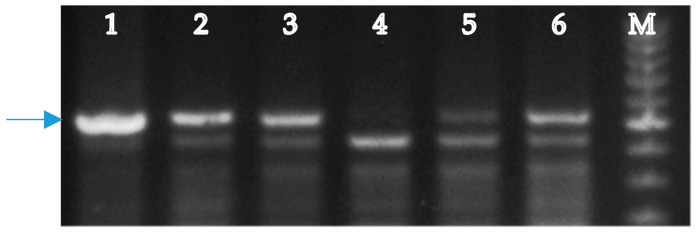
PCR amplification pattern of the marker scs265 linked to gene *Lr19*. Lanes: 1, *Ag. elongatum* (control); 2, 311(4) × Om37; 3, L-311(4) × L-134; 4, Pyr28; 5-6, Om37; M, 100 bp DNA ladder. Arrow indicates the 512-bp band specific for *Ag. elongatum*.

A molecular analysis of fertile plants (2n = 42) from groups I and II was performed to detect the presence of genes *Lr26* and *Sr31* in R1 plants of hybrid combinations L-311(4) × Om37, L-311(4) × L-134, and L-311(4) × 2870, and genes *Lr19* and *Sr25* in R1 plants of hybrid combinations L-311(4) × Om37 and L-311(4) × L-134. The marker iag95 amplified a band of 1100 bp in rye *S. cereale* and L-311(4), Om37, L-311(4) × Om37, L-311(4) × L-134, L-311(4) × 2870, but no band amplified in Pyr 28 (Figure 3), indicating that variety Pyr28 does not carry the gene *Lr26.* Based on the amplification of the 1100 bp band fragment, the variety Omskaya 37 and L-311(4), L-311(4) × Om37, L-311(4) × L-134, L-311(4) × 2870 were identified to carry *Lr26.* The marker scs265 amplified a band of 512 bp in *Ag. elongatum* and 311(4) × Om37, L-311(4) × L-134, Om37 (Figure 4) showing these genotypes carrying the gene *Lr19*. No product was obtained in Pyr28. 

**Figure 5:**
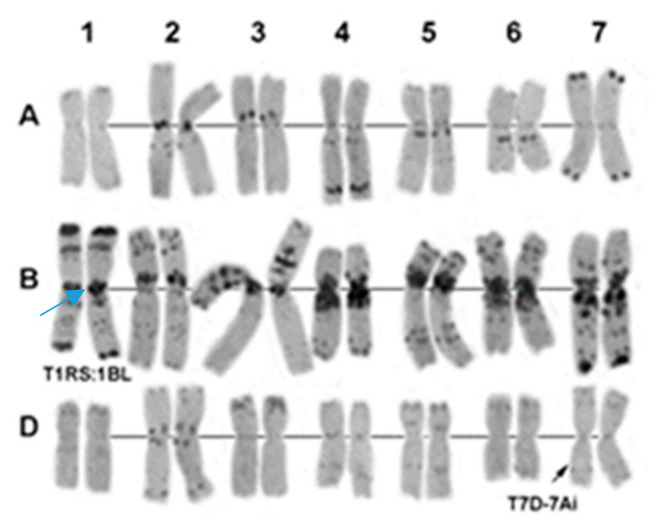
The results of C-banding of chromosomes of the androgenic R1 of hybrid combination L-311(4) × Om37. 1–7, homeologous groups. The arrows indicate wheat–rye T1RS.1BL and wheat–wheatgrass T7D-7Ai translocations.

C-banding of hybrid combinations L-311(4) × Om37 and L-311(4) × L-134, in which a combination of the *Lr26*/*Sr31* and *Lr19*/*Sr25* genes was revealed, confirmed the presence of wheat–rye 1RS.1BL and wheat–wheatgrass 7DL-7Ai translocations (Figure 5).

### 2.5. Evaluation of Agronomic Parameters and Resistance to Fungal Pathogens in Alloplasmic Sister DH Lines of Three Hybrid Combinations 

Fourteen alloplasmic sister DH lines from three families of hybrid combination L-311(4) × Om37, nine DH lines from three families of hybrid combination L-311(4) × L-134, and eleven DH lines from three families of hybrid combination L-311(4) × 2870 were selected for field trials in 2016. Table 7 shows the data presence combinations of the resistance genes to leaf rust (*Lr26* and *Lr19*) and stem rust (*Sr31* и *Sr25*), localized on wheat–alien translocations 1RS.1BL and 7DL-7Ai, in plants R1, which are the source of sister alloplasmic DH lines. In addition, this table shows the number of plants of DH lines that have reached the heading per the number of seeds sown, as well as the results of studying resistance to leaf rust, stem rust, and powdery mildew.

It was established that the control variety Omskaya 33 was highly susceptible to leaf rust and stem rust and susceptible to powdery mildew. This indicates a strong distribution of these fungal pathogens in the year of studying DH lines. All alloplasmic sister DH lines of three families of hybrid combination L-311(4) × Om37, like the parent euplasmic sister line Om37, carry combinations of the genes *Lr26*/*Sr31* and *Lr19*/*Sr25* and had the same high level of resistance to leaf rust and stem rust. These sister DH lines exceeded the level of resistance in the maternal L-311(4) line (with genes *Lr26*/*Sr31*) which was immune only to stem rust. They were either moderately resistant or moderately susceptible, as were the parent lines—the alloplasmic maternal line L-311(4) and the euplasmic paternal line Om37. Five of the six sister DH lines of the family 3 had lower rates of the number of plants that reached heading per the number of seeds sown compared to the control. To avoid possible aneuploidy, all DH lines of this family were excluded from further field trials.

Nine alloplasmic sister DH lines of three families of hybrid combination L-311(4) × L-134, carrying combinations of the genes *Lr26*/*Sr31* and *Lr19*/*Sr25* may also have a powdery mildew resistance gene *Pm4b*, since the wheat variety Reno, a carrier of this gene, is present in the L-134 pedigree [51]. Only the sister DH lines from the family 3 of this hybrid combination, along with immunity to leaf rust and stem rust, had resistance to powdery mildew and exceeded the level of resistance to leaf rust and powdery mildew in the maternal line L-311(4), and exceeded the level of resistance to powdery mildew in control line Om37. The DH lines from family 1 and family 2 of hybrid combination L-311(4) × L-134 showed either moderate resistance or moderate susceptibility to powdery mildew (Table 7) and were excluded from further field trials.

Eleven alloplasmic sister DH lines of hybrid combination L-311(4) × 2870, carrying the gene combination *Lr26*/*Sr31*, may have another resistance gene to leaf rust, as well as a resistance gene to powdery mildew, since the paternal line 2870 carries these genes introgressed from *T. dicoccoides* [52]. An analysis of resistance to fungal pathogens revealed four sister DH lines of family 1 of hybrid combination L-311(4) × 2870 that were immune to leaf rust, stem rust, and powdery mildew. The sister DH lines of family 2 and family 3 did not show high resistance to leaf rust and powdery mildew, which could be due to the loss of genetic material from *T. dicoccoides*. DH lines from these families were not studied later in breeding work.

Alloplasmic sister DH lines of two families of hybrid combinations L-311(4) × Om37, one family of hybrid combinations L-311(4) × L-134, and one family of hybrid combination L-311(4) × 2870 were tested in 2017. Table 8 shows the results of studying some agronomic characteristics and resistance to fungal pathogens in these DH lines. As in 2016, the alloplasmic sister DH lines of family 1 and family 2 of the hybrid combination L-311 (4) × Om37 were at the level of paternal euplasmic line L-37 in terms of resistance to fungal pathogens and exceeded the resistance to leaf rust and powdery mildew in the maternal alloplasmic line L-311(4). However, the sister lines of family 1 of this hybrid combination had a longer heading date than the sister lines of family 2 (Table 8) and were excluded from further work. Alloplasmic sister DH lines of the family 2 of hybrid combination L-311 (4) × Om37 were at the level of the parent genotypes according to agronomic indicators such as yield, 1000-grain weight, and protein content.

Two alloplasmic sister lines of the hybrid combination L-311(4) × L-134 and the four sister lines of the hybrid combination L-311(4) × 2870 were also at the level of the alloplasmic maternal line L-311 (4) in terms of agronomic indicators but exceeded the maternal line in resistance to leaf rust and powdery mildew.

In the next two years of field trials (2018 and 2019), there was also a strong spread of fungal pathogens. This conclusion was made from observations of the susceptible control variety Omskaya 33, which was strongly affected by leaf rust and stem rust and was susceptible to powdery mildew (Table 9 and Table 10). Against this background, alloplasmic sister DH lines of the hybrid combination L-311 (4) × Om37 showed resistance to leaf rust and stem rust and moderate resistance or moderate susceptibility to powdery mildew. Alloplasmic sister DH lines of hybrid combinations L-311(4) × L-134 and L-311 (4) × 2870 showed immunity or resistance to leaf rust, stem rust, and powdery mildew during these years of field trials. In alloplasmic sister DH lines of all three hybrid combinations, the values of agronomic indicators were at the level of the parent genotypes, or exceeded them.

Alloplasmic sister DH lines within each family were characterized by almost identical indicators of resistance to fungal pathogens. According to 2019 data (Table 10), differences between alloplasmic sister lines R17-DH1–R17-DH4 of the hybrid combination L-311(4) × Om37 were observed for yield values and protein content. Yield values are significantly higher compared to other sister DH lines in the R17-DH4 line, and protein content values are significantly higher in the R17-DH1 and R17-DH3 lines than in the R17-DH2 and R17-DH4 sister lines. The yield of the R17-DH4 line exceeded both parent genotypes, L-311(4) and Om37. The protein content of sister lines R17-DH1–R17-DH4 was either at the level of the parent lines (R17-DH2 and R17-DH2) or higher (R17-DH1 and R17-DH3). The value of 1000-grain weight of those sister lines was at the level of the parent lines.

Alloplasmic sister lines R28-DH1 and R28-DH2 of the hybrid combination L-311(4) × L-134 were similar in terms of heading date, yield, and protein content. However, the 1000-grain weight value of the R28-DH2 line was higher than that of its sister line R28-DH1. The R28-DH1 and R28-DH2 lines did not differ in yield from the maternal line L-311 (4), their 1000-grain weight was at the level of L-311(4) (R28-DH1 line) or exceeded it (R28-DH1 line), and the protein content was at the level of L-311(4).

Alloplasmic sister lines R51-DH1–R51-DH4 of the hybrid combination L-311(4) × 2870 had similar values for all studied characteristics, including immunity to fungal pathogens. Agronomic indicators of these sister lines were at the level of the maternal line L-311(4), and their resistance to leaf rust and powdery mildew significantly exceeded maternal line L-311(4).

## 3. Discussion

Androgenesis and the formation of families of regenerants in anther culture in alloplasmic (*H. vulgare*)–*T. aestivum* and euplasmic genotypes and their hybrids were studied. It was shown that parameters of androgenesis such as the number of embryo-like structures and the total number of regenerants and green regenerants per 100 anthers varied depending on the genotype. Among the studied genotypes, the lowest values of these parameters were in the euplasmic lines Om37 and Om38 and the alloplasmic recombinant line L-17(3). The highest values of the three studied indicators of androgenesis were in the alloplasmic line L-311(4), carrier of translocation 1RS.1BL. These results were consistent with our previously obtained data in a study of androgenesis of other lines isolated from varieties Omskaya 37 and Omskaya 38 and the hybrid population L-311/00-22 in anther culture [48,53]. The high androgenesis rates in the L-311(4) line can be explained by the presence of the 1RS.1BL translocation. It is known that in anther culture of bread wheat, depending on the genotype, the 1RS.1BL translocation can have a positive effect on the formation of embryoids and the development of seedlings [54,55], including green seedlings [55].

The Om37 and Om38 lines, with low values of androgenesis, carried wheat–rye 1RS.1BL and wheat–wheatgrass 7DL-7Ai translocations. In some bread wheat genotypes with 7DL-7Ai translocations, the formation of androgenic embryoids and the regeneration of green seedlings are suppressed [56]. In other genotypes of wheat with this translocation, only increased green plant regeneration has been observed [44]. Thus, in the presence of the wheat–rye 1RS.1BL translocation, the negative effect of the 7DL-7Ai translocation on androgenesis dominated in the Om37 and Om38 lines. However, in the genetic background of hybrids L-311(4) × Om37, L-311(4) × L-134, and L-311(4) × Om38, the negative effect of the 7DL-7Ai translocation on androgenic ability in anther culture did not occur in the presence of the wheat–rye 1RS.1BL translocation. In these hybrids, the values of the studied indicators of androgenesis are significantly higher than in the Om37 and Om38 lines. In the hybrid combination L-311(4) × 2870, in addition to the 1RS.1BL translocation, there is genetic material from the 2870 line, the pedigree of which includes *T. dicoccoides* [52]. The number of green plantlets/100 anthers in the hybrid combination L-311(4) × 2870 was significantly lower than that of hybrids L-311(4) × Om37 and Om37 × L-311(4). These results show that the genotype significantly affects the efficiency of anther culture, which is consistent with the data of other works [43,44,56]. Moreover, as discussed in [44,57], in vitro androgenic response could be transferred from a variety with the ability to regenerate in its F1 hybrids.

It was noted that in the L-17(3) line and hybrids L-311(4) × Om37 and L-311(4) × 2870, the number of all regenerants per 100 anthers was higher than the number of ELS/100 anthers. This is because most often, one ELS did not produce single plantlets, but clusters (families) of regenerants that developed from polyembryos. This concerned both albino and green plantlets. With regard to the analysis of green plantlet development, it has been observed that regardless of the genotype, families of regenerants are formed rather than single plantlets. The formation of androgenic polyembryos was noted in anther culture of bread wheat [58] and triticale [50]. Inducers of polyembryoid formation include 2,4-D present in the induction medium [58].

It is also possible that the development of families of multiple regenerants depends on the genotype. The present work noted a tendency for the number of green plantlets to increase in families of alloplasmic genotype L-311(4), and some of its alloplasmic hybrids in comparison with euplasmatic lines Pyr28 and Om37. It can be assumed that in alloplasmic genotype (*H. vulgare*)–*T. aestivum* carrying the 1RS.1BL translocation, increased induction of polyembryos was the result of the mutual influence of barley cytoplasm and rye chromosome 1RS. This is consistent with the presence of polyembryony and the development of twins from the seeds of F1 hybrids obtained from pollination of (*H. vulgare*)–*T. aestivum* lines with pollen of wheat–rye substitution lines 1R(1A) and 1R(1D) [59]. The development of twins in the alloplasmic lines of bread wheat variety Salmon carrying the cytoplasm of *Aegilops kotschyi* Boiss. and rye chromosome 1RS has been described previously [60].

In our work, the level of fertility of regenerants within families was studied. Approximately half of the green regenerant families (46.82%) were found to have all sterile sister plants. The frequency of families in which all sister plants were partially fertile was 9.09%, all plants were fertile in 20% of families, and 24.09% of families consisted of regenerant clusters at different fertility levels. The presence of such families can be explained by the participation of several polyembryoids in their formation. Thus, in 75.91% of families, regenerants with identical fertility were found. We assumed that fertility is not an accurate measure of kinship between sister lines within the same family. However, for a quick selection of required families, this method can be convenient. In this work, the frequency of families with green R0 regenerants identical in fertility was not significantly distinguishable from the value revealed in the study of androgenic families of triticale, which showed that in about 80% of cases, all members of a family were genetically identical using DNA markers [50].

In our work, the average percentage of R0 regenerants that restored fertility varied depending on genotype and was 37.74%. Among the families of R1 sister lines grown from the seeds of individual fertile R0 spikes of the alloplasmic line L-311(4) and its three hybrid combinations (L-311(4) × Om37, L-311(4) × L-134, and L-311(4) × 2870), three groups were identified that differed in the level of plant fertility. On average, only 50% of families had sister lines, all of whose plants were fertile. In two other groups of families with different frequency, the sister lines segregated for fertile, partially fertile, and sterile plants. The frequency of families with plant fertility segregation was higher for hybrid combinations compared to their maternal line L-311(4). Cytogenetic analysis showed that the majority of plants with reduced fertility were aneuploids, which is consistent with our previous data [48,49] and data of other authors [41,44]. In this regard, for field trials, sister DH lines of families of hybrid combinations L-311(4) × Om37, L-311(4) × L-134, and L-311(4) × 2870 were formed only from seeds of the 42-chromosome plants of individual R1 sister lines with full fertility (more than 30 seeds in the main spike) carrying target genes localized in wheat–alien translocations.

Fourteen alloplasmic sister DH lines from three families of hybrid combination L-311(4) × Om37, nine DH lines from three families of hybrid combination L-311(4) × L-134, and eleven DH lines from three families of hybrid combination L-311(4) × 2870 were selected for field trials. By the fourth year of testing, three families of sister DH lines of three hybrid combinations were selected and studied. It was established that in terms of resistance to fungal pathogens, alloplasmic sister DH lines within families did not differ, showing the same level of resistance as in parental genotypes, or higher. The increased resistance to leaf rust and powdery mildew in sister DH lines compared to the maternal alloplasmic line L-311(4) indicates that new resistance genes from donor lines used as paternal genotypes during hybridization were introgressed into the genetic background of this line. It can be assumed that the resistance to powdery mildew in sister lines R28-DH1 and R28-DH2 of the hybrid combination L-311(4) × L-134 is due to the influence of the *Pm4b* gene, the carrier of which is the variety Reno [60], which is part of the L-134 pedigree. High resistance (immunity) to leaf rust and powdery mildew in sister lines R51-DH1-R51-DH4 of the hybrid combination L-311(4) × 2870 were transmitted from their paternal line 2870, which carries these genes introgressed from *T. dicoccoides* [55]. High resistance to leaf rust in sister lines R17-DH1 – R17-DH4 can be explained by the mutual influence of genes *Lr 26* and *Lr19*, and high resistance to stem rust by mutual influence of genes *Sr31* and *Sr25* due to the presence of wheat–rye 1RS.1BL and wheat–wheatgrass 7DL-7Ai translocations, respectively [61].

The high susceptibility of the Omskaya 33 variety to leaf rust and stem rust, and its susceptibility to powdery mildew, shows that in the year of testing there was a strong spread of fungal pathogens. Thus, the infectious background was strongly pronounced, which makes it possible to objectively judge the resistance of DH lines to leaf rust, stem rust, and powdery mildew. According to agronomic characteristics, sister DH lines were either at the level of parental genotypes or exceeded them. The studied DH lines are promising for further breeding work.

## 4. Materials and Methods

### 4.1. Plant Material

In this work, we used alloplasmic and euplasmic genotypes and hybrids between them. Alloplasmic recombinant line L-17(3) originated from the fertile progeny of the BC3 generation of a barley–wheat hybrid (*H. vulgare*, var. Nepolegaushii × *T. aestivum*, var. Saratovskaya 29) [15]. Alloplasmic line L-311(4) carried the 1RS.1BL translocation and was a progeny of a hybrid between DH(1) of alloplasmic line L-17(3) and euplasmic line Com37 having 1RS.1BL translocation [15]. Two euplasmic lines, Om37 and Om38, were used for anther culture as controls and to produce hybrids with alloplasmic line L-311(4). These lines were isolated from varieties of spring bread wheat of West Siberian breeding, Omskaya 37 and Omskaya 38, which carry wheat–rye 1RS.1BL and wheat–wheatgrass 7DL-7Ai translocations (7Ai from *Agropyron elongatum*) [61]. The reciprocal hybrid F1 between L-311(4) and Om37, and hybrids L-311(4) × Om38, L-311(4) × L-134, and L-311(4) × 2870, were used. Pedigree of L-134 is Omskaya 21/Reno//Omskaya 33/Omskaya 37. Line 2870 was obtained by crossing Saratovskaya 55 with *T. dicoccoides* [52]. As a control, we used a euplasmic line of the Pyrotrix 28 (Pyr28) variety of spring bread wheat, which does not carry wheat–alien translocations. The donor plants of each genotype for anther culture were grown in the greenhouse of the Institute of Cytology and Genetics SB RAS in autumn–winter 2014.

### 4.2. Anther Culture and Plant Regeneration

Spikes of primary tillers were harvested from donor plants when most of the microspores were at the mid to late uninucleate stage. Spikes were kept in flasks in a 25% solution of macronutrients of Gamborg B5 medium [62] and put in the dark at 40 C for 4 to 9 days. Before anther excision, the surfaces of the spikes were sterilized with 70% ethanol. Anthers were transferred onto the P-II medium [63], supplemented with 0.75 mg/L of 2.4-D, sucrose (45 g/L), maltose (45 g/L), and Bacto Difco agar (8 g/L). For cultivation, 100 mL mL beaker with 25 mL mL medium were used. The beakers were covered with foil lids, which were sealed with parafilm. The anthers were incubated in the dark at 29 °C. All the developed embryo-like structures with a diameter of about 1 mm were transferred to the Gamborg medium (B5) [62] with 30 g/L saccharose without growth regulators. The embryo-like structures were incubated for 3–4 weeks in the culture room (16/8 h light/dark) at 24 °C.

### 4.3. Acclimatized Anther Culture-Derived Regenerants

Developed green regenerants at the 3-leaf stage were transferred from the medium to Petri dishes with water. As the plates were shaken, the agar residues were washed and clusters (families) of regenerants and single regenerants were separated from each other. Individual seedlings and their clusters were planted in 500 mL vegetation vessels containing fine clay pellets and covered with glass to maintain the moisture content. For 3 weeks, the seedlings were fed with Gamborg B5 [62] liquid medium without vitamins, half diluted with water. After acclimatization for 3 weeks, single regenerants and their families were transplanted together with soil into 3000 mL vegetation vessels. For proper plant growth, required fertilizers were applied, alternating with water.

### 4.4. Analysis of the Results

The 3 important parameters of in vitro androgenesis—number of embryo-like structures (ELS), number of all regenerants/100 anthers, and number of green plantlets/100 anthers—were analyzed. All parameters are expressed as percentages. The efficiency of the development of green regenerant families from ELS compared to single green regenerants was studied, comparing the following indicators: single green plantlets/100 ELS, families of green plantlets/100 EPS, average number of green plantlets per family, and variation of green plantlets in one family. Families of green plants were characterized by fertility level during harvesting in May 2015: sterile (S)—no seed set and set of seeds from 1 to 2 in the spike; partly fertile (PF)—set of seeds from 3 to 15 in the spike; or fertile (F)—set of seeds over 15 grains. The percentage of spontaneous rediploidization was estimated, taking into account all partly fertile and fertile androgenic plants.

The seeds of each individual spike in fertile R0 plants that set at least 20 seeds were used to form sister R1 lines of families of 4 alloplasmic genotypes: L-311(4) and its hybrids L-311(4) × Om37, L-311(4) × L-134, and L-311(4) × 2870. Twenty plants of each sister R1 line were grown in a hydroponic greenhouse in autumn–winter 2015 and winter–spring 2016. In these R1 sister lines, the level of fertility according to the number of seeds in the main spike was assessed, and a cytogenetic analysis was performed. Individual fertile plants of 42-chromosome R1 sister lines of hybrid combination L-311(4) × 2870 were tested for the presence of the wheat–rye 1RS.1BL translocation, and hybrid combinations L-311(4) × Om37 and L-311(4) × L-134 were tested for the presence of the wheat–rye 1RS.1BL and wheat–wheatgrass 7DL-7Ai translocations using molecular analysis and C-banding.

### 4.5. Cytological and Molecular Analysis

An analysis of chromosome number was carried out using Feulgen staining. The tips of the roots for analysis were collected from plants grown in a hydroponic greenhouse according to previously used methods [48]. The C-banding technique was performed according to the protocol described by Badaeva et al. [64]. SCAR-marker iag95, linked to the genes *Lr26* and *Sr31* and localized on the short arm of the rye chromosome 1R, was used to confirm the presence of the 1RS.1BL translocation in the initial lines, hybrids, and regenerants derived from these lines [65]. The SCAR-marker scm265, linked with the gene *Lr19* and localized on the *Ag. elongatum* (Host.) Beauv chromosome 7AgL, was used to identify the wheat–wheatgrass 7DL-7Ai translocation [66].

### 4.6. Field Studies of Alloplasmic Sister DH Lines

For field studies, alloplasmic sister DH lines of hybrid combinations L-311(4) × Om37, L-311(4) × L-134, and L-311(4) × 2870 were formed from seeds of the 42-chromosome plants of individual sister lines with full fertility (more than 30 seeds in the main spike) carrying target genes localized on wheat–alien translocations. Fourteen DH lines from 3 families of L-311(4) × Om37, 9 DH lines from 3 families of L-311(4) × L-134, and 11 DH lines from 3 families of L-311(4) × 2870 have been included in the breeding program of the Omsk Agrarian Scientific Center since 2016. These DH lines, grown in breeding nurseries, were studied. Methods of growing and studying lines, including agronomic characteristics (heading data, yield, and protein content in the grain) were carried out according to the previously described methods [61]. Resistance to leaf rust (*Puccinia recondita* f. sp. *tritici*), stem rust (*Puccinia graminis* f. sp. *tritici*), and powdery mildew (*Blumeria graminis* f. sp. *tritici*) were estimated during the growing season 4 times in 5–6 days, starting with the appearance of symptoms of the disease. To assess the degree of damage, a scale based on infection types was used, 0–100% coverage of leaves with leaf rust or powdery mildew and stem and leaves with stem rust according to main modifications of Cobb’s [67,68] scales. According to these scales: 0 = immune, 1–10% = resistance (R), 11–30% = moderately resistance (MR), 31–50% = moderately susceptible (MS), 51–60% = susceptible (S), 61% and above = highly susceptible (HS). In 2016, 45 seeds of each line were sown on an area of 0.25 m^2^. Families of sister lines for further work were selected based on data on resistance to fungal pathogens and the number of plants of DH lines that have reached the heading per the number of seeds sown (%). In 2017, each sister DH line was grown on an area of 5 m^2^; in 2018 and 2019, on an area of 10 m^2^. The controls were variety Omskaya 33 (susceptible to fungal pathogenes) and alloplasmic line L-311(4) and euplasmic line Om37, which are the parent genotypes of the studied alloplasmic sister DH lines. Molecular analysis of samples of DH lines was carried out to confirm the presence of wheat–alien translocations.

### 4.7. Statistical Analyses

For each genotype, at least 300 anthers were cultivated in one experiment, placing approximately 30 anthers from one spike on the surface of the medium of one beaker. Every experiment was repeated at least three times. Field studies were conducted in three areas of the Omsk region. The data were statistically evaluated by one-way ANOVA and Student’s t-test.

## 5. Conclusions

It was shown that the values of parameters of androgenesis such as number of embryo-like structures and number of all regenerants and green regenerants/100 anthers varied depending on genotype. Despite the different responses of genotypes to cultivation conditions, all of them are characterized by a common feature, the predominant development of families of regenerants originating from polyembryoids rather than single regenerants. Families can be ranked by the level of fertility of regenerants, or family members. However, families that differ in fertility and ploidy can develop as single mixed clusters, which is associated with the participation of several embryo-like structures in their formation. In order for DH lines not to be populations of different genotypes and not to include aneuploids, they must be formed from the most productive 42-chromosomal plants R1, which are grown from seeds set in a separate fertile spike of regenerants R0. Pre-breeding should include cytogenetic and molecular analysis to prove the presence of introgressed genes. This work shows the possibility of obtaining novel introgression DH lines with complex resistance to fungal pathogens with the involvement of alloplasmic genotypes (*H. vulgare*)–*T. aestivum* with fertility restoration and fixed nuclear–cytoplasmic compatibility.

## 6. Patents

Patent of the Russian Federation for breeding achievement No. 7950. Spring bread wheat (*Triticum aestivum* L.) var. Sigma. Registered in the State Register of Protected Breeding Achievements 08.24.2015.

Patent of the Russian Federation for breeding achievement No. 9568. Spring bread wheat (*Triticum aestivum* L.) var. Uralosibirskaya 2. Registered in the State Register of Protected Breeding Achievements 14.03.2018.

Patent of the Russian Federation for breeding achievement No. 10854. Spring bread wheat (*Triticum aestivum* L.) var. Ishimskaya 11. Registered in the State Register of Protected Breeding Achievements 31.01.2020.

## Figures and Tables

**Table 1:** Efficiency of anther culture in four euplasmic (Pyr28; Om37; Om38; Om37 × L-311(4)) and six alloplasmic (L-17(3); L-311(4); L-311(4) × Om37; L-311(4) × L-134; L-311(4) × Om38; L-311(4) × 2870) genotypes. Different letters within the same column indicate statistically significant differences (*p* < 0.05). ELS, embryo-like structures.

Genotype	Number of ELS/100 Anthers	Number of all Plantlets/100 Anthers	Number of Green Plantlets/100 Anthers
Pyr28	57.53 ab	29.23 ab	7.00 a
Om37	24.76 a	12.20 a	5.06 a
Om38	8.93 a	3.16 a	0.20 a
L-17(3)	10.76 a	16.60 a	9.86 a
L-311(4)	124.46 c	105.20 c	61.76 c
L-311(4) × Om37	65.51 b	78.03 b	42.73 bc
Om37 × L-311(4)	67.63 b	60.33 b	39.20 b
L-311(4) × L-134	85.43 b	36.76 ab	20.33 ab
L-311(4) × Om38	69.77 b	47.70 b	19.46 ab
L-311(4) × 2870	46.05 ab	51.23 b	14.39 a
Mean	56.08	44.04	21.99
LSD5% =	18.70	11.76	10.29

**Table 2:** Efficiency of developing green plantlet (GP) families compared to single GPs as a result of EPS cultivation, average number of GPs per family, and GP variation in one family. Different letters within the same line indicate statistically significant differences (*p* < 0.01, Student’s t-test). ns, not significant. Euplasmic genotypes: Pyr28, Om37, Om38, Om37 × L-311(4); alloplasmic genotypes: L-17(3), L-311(4), L-311(4) × Om37, L-311(4) × L-134, L-311(4) × Om38, L-311(4) × 2870.

Genotype	Number of Single GPs/100 EPS (Mean ± SE)	Number of GP Families/100 EPS (Mean ± SE)	Number of GPs/Family	GP Variation in One Family
Pyr28	1.17 ± 0.33 a	2.75 ± 0.51 b	4.35	2–10
Om37	1.00 ± 0.44 a	3.40 ± 0.81 b	3.70	3–12
Om38	0	2.27 ± 1.58 ns	2.00	–
L-17(3)	1.20 ± 0.84 a	7.22 ± 2.00 b	5.61	2–22
L-311(4)	0.95 ± 0.22 a	5.11 ± 0.52 b	8.83	2–31
L-311(4) × Om37	0.83 ± 0.37 a	5.68 ± 0.94 b	9.91	2–36
Om37 × L-311(4)	1.83 ± 0.57 a	8.42 ± 1.18 b	6.78	2–20
L-311(4) × L-134	0.79 ± 0.29 a	3.84 ± 0.64 b	5.00	3–25
L-311(4) × Om38	1.91 ± 0.57 a	7.15 ± 1.07 b	5.14	3–28
L-311(4) × 2870	1.01 ± 0.41 a	7.89 ± 1.11 b	6.75	2–35
Medium	1.07 ± 0.12 a	5.37 ± 0.27 b	5.81	2–36

**Table 3:** Characterization of families of green R0 plants of three euplasmic (Pyr28; Om37; Om37 × L-311(4) and six alloplasmic (L-17(3); L-311(4); L-311(4) × Om37; L-311(4) × L-134; L-311(4) × Om38; L-311(4) × L-2870) genotypes by fertility: sterile (S), partly fertile (PF), fertile (F).

Genotype	Number of Families with Green Plants	Frequency (%) Families with Androgenic Plants with Certain Level of Sterility/Fertility			
		S	PF	F	S + F and S + PF + F
Pyr28	11	63.64	9.09	18.18	9.09
Om37	12	41.66	8.33	16.68	33.33
L-17(3)	13	61.54	15.38	7.70	15.38
L-311(4)	45	57.78	8.89	15.55	17.78
L-311(4) × Om37	20	45.00	10.00	25.00	20.00
Om37 × L-311(4)	34	52.94	8.82	17.65	20.59
L-311(4) × L-134	18	44.44	5.56	27.78	22.22
L-311(4) × Om38	20	55.00	10.00	5.00	30.00
L-311(4) × 2870	47	23.40	8.51	38.30	29.79
Mean	220	46.82	9.09	20.00	24.09

**Table 4:** Percentage of spontaneous rediploidization in partially fertile and fertile R0 plants from families of androgenic regenerants of three euplasmic (Pyr28, Om37, Om37 × L-311(4)) and six alloplasmic (L-17(3), L-311(4), L-311(4) × Om37, L-311(4) × L-134, L-311(4) × Om38, L-311(4) × L-2870) genotypes.

Genotype	No. of Tested Plants	No. of Partially Fertile and Fertile Plants (DH)	Frequency (%) of Spontaneous Rediploidization
Pyr28	48	14	29.17
Om37	44	10	22.72
L-17(3)	86	38	44.18
L-311(4)	297	136	45.79
L-311(4) × Om37	198	67	33.83
Om37 × L-311(4)	174	40	22.98
L-311(4) × L-134	85	20	23.52
L-311(4) × Om38	90	17	18.88
L-311(4) × L-2870	292	154	52.73
Mean	1314	496	37.74

**Table 5:** Fertility level in R1 sister lines (R1SL) of four alloplasmic genotypes. Different letters within the same column indicate statistically significant differences (*p* < 0.05, Student’s t-test).

Genotype	No. of Families Tested	Frequency (%) of Families		
		Group I	Group II	Group III
		100% Fertile Plants in R1SL	75–95% Fertile Plants in R1SL	Less than 50% of Fertile Plants in R1SL
L-311(4)	15	80.00 a	13.33 b	6.67 b
L-311(4) × Om37	10	40.00 ab	40.00 ab	20.00 ab
L-311(4) × L-134	8	37.50 ab	25.00 ab	37.50 ab
L-311(4) × 2870	19	36.84 b	47.37 a	15.79 ab
Mean of hybrid	37	37.84 b	40.54 a	21.62 a
Mean	52	50.00	32.69	17.31

**Table 6:** Ploidy level of fertile and partly fertile R1 sister plants of four alloplasmic genotypes. The difference between the frequency of hexaploids and aneuploids in each group of fertile and partially fertile plants is significant at *p* = 0.001.

Genotype	Total Green Plants R_1_ Tested	Plants with Ploidy Level			
		Hexaploids		Aneuploids	
		No.	Frequency (%)	No.	Frequency (%)
	**Fertile Plants from Group I and Group II**				
L-311(4)	39	37	94.87	2	5.13
L-311(4) × Om37	31	28	90.32	3	9.68
L-311(4) × L-134	24	22	91.67	2	8.33
L-311(4) × 2870	48	41	85.42	7	14.58
Mean	142	128	90.14	14	9.86
	**Partly Fertile Plants from Group II and Group III**				
L-311(4)	14	2	14.29	12	85.71
L-311(4) × Om37	10	0	0	10	100
L-311(4) × L-134	11	1	9.09	10	90.91
L-311(4) × 2870	15	4	26.67	11	73.33
Mean	50	7	14.00	43	86.00

**Table 7:** The presence of resistance genes to fungal pathogen in plants R1, sources of alloplasmic sister DH lines, number of plants of DH lines reached heading per number of seeds sown (%), and resistance to fungal pathogens in alloplasmic sister DH lines of three hybrid combinations in 2016. Controls: variety Omskaya 33, lines Om37 and L-311(4). (0: immune; R: resistant; MR: moderately resistant; MS: moderately susceptible; S: susceptible; HS: highly susceptible). Different letters within the same column indicate statistically significant differences (*p* < 0.05, Student’s t-test).

Genotype, Families, Sister DH lines	The Presence of Fungal Pathogen Resistance Genes in Plants R1, Sources of DH Lines		Number of Plants of DH Lines Reached Heading/Number of Seeds Sown (%)	Resistance to Fungal Pathogens		
	*Lr26*/*Sr31*	*Lr19*/*Sr25*		Leaf Rust	Stem Rust	Powdery Mildew
**L-311(4) × Om37**						
**Family 1**						
R16-DH1	+	+	84.44 b	0	0	MS
R16-DH2	+	+	82.22 b	R	0	MR
R16-DH3	+	+	86.67 b	0	0	MR
R16-DH4	+	+	88.89 b	0	0	MS
						
**Family 2**						
R17-DH1	+	+	84.44 b	0	0	MS
R17-DH2	+	+	88.89 b	0	0	MR
R17-DH3	+	+	75.55 b	R	0	MS
R17-DH4	+	+	86.67 b	0	0	MR
**Family 3**						
R18-DH1	+	+	46.67 a	R	0	MS
R18-DH2	+	+	51.11 a	0	0	MS
R18-DH3	+	+	44.44 a	0	0	MS
R18-DH4	+	+	48.89 a	0	0	MS
R18-DH5	+	+	42.22 a	0	0	MS
R18-DH6	+	+	73.33 b	R	R	MS
**L-311(4) × L-134**						
**Family 1**						
R26-DH1	+	+	82.22 b	R	0	MS
R26-DH2	+	+	86.67 b	R	0	MS
R26-DH3	+	+	84.44 b	0	0	MR
R26-DH4	+	+	80.00 b	R	0	MS
**Family 2**						
R27-DH1	+	+	82.22 b	0	0	MR
R27-DH2	+	+	73.33 b	0	0	MS
R27-DH3	+	+	75.55 b	0	0	MS
**Family 3**						
R28-DH1	+	+	86.67 b	0	0	R
R28-DH2	+	+	88.89 b	0	0	R
**L-311(4) × 2870**						
**Family 1**						
R51-DH1	+	-	88.89 b	0	0	0
R51-DH2	+	-	84.44 b	0	0	0
R51-DH3	+	-	86.67 b	0	0	0
R51-DH4	+	-	88.89 b	0	0	0
**Family 2**						
R52-DH1	+	-	82.22 b	MS	0	MS
R52-DH2	+	-	77.77 b	MS	0	MS
R52-DH3	+	-	84.44 b	MS	0	MS
**Family 3**						
R53-DH1	+	-	82.22 b	MS	0	MS
R53-DH2	+	-	86.67 b	MS	0	MS
R53-DH3	+	-	84.44 b	MS	0	MS
R53-DH4	+	-	80.00 b	MS	0	MS
**Controls**						
Omskaya 33	-	-	80.00 b	HS	HS	S
Om37	+	+	86.66 b	0	0	MS
L-311(4)	+	-	88.88 b	MS	0	MS

**Table 8:** Values of agronomic parameters and resistance to fungal pathogens in alloplasmic sister DH lines of three families of three hybrid combinations in 2017. Controls: variety Omskaya 33, lines Om37 and L-311(4). (0: immune; R: resistant; MR: moderately resistant; MS: moderately susceptible; S: susceptible; HS: highly susceptible). Different letters within the same column indicate statistically significant differences (*p* < 0.05).

Genotype and Sister DH Lines	Heading Date	Yield (t/ha)	1000-Grain Weight, (g)	Protein Content, (%)	Resistance to Fungal Pathogens		
					Lea fRust	Stem Rust	Powdery Mildew
**L-311(4) × Om37**							
**Family 1**							
R16-DH1 DH1	102 c	3.01 ab	-	-	0	0	MS
R16-DH2	99 c	3.12 ab	-	-	0	0	MS
R16-DH3	100 c	2.60 a	-	-	R	0	MR
R16-DH4	98 c	2.98 ab	-	-	0	0	MS
**Family 2**							
R17-DH1	90 a	4.14 ab	36.00 bc	16.53 c	0	R	MR
R17-DH2	89 a	4.87 ab	35.45 bc	16.02 bc	R	0	MS
R17-DH3	91 a	4.46 ab	38.40 bc	17.13 c	0	R	MS
R17-DH4	90 a	4.90 ab	37.65 bc	16.13 bc	0	0	MR
**L-311(4) × L-134**							
R28-DH1	94 b	4.15 ab	42.40 c	13.96 b	0	0	MR
R28-DH2	96 bc	4.30 ab	37.20 bc	14.00 b	0	0	MR
**L-311(4) × 2870**							
R51-DH1	93 ab	4.94 ab	39.50 c	14.42 bc	0	0	0
R51-DH2	92 ab	5.03 b	39.50 c	15.76 bc	0	0	0
R51-DH3	94 b	5.14 b	36.80 bc	14.31 bc	0	0	0
R51-DH4	95 bc	4.89 ab	39.40 c	16.13 bc	0	0	0
**Controls**							
Omskaya 33	94 b	3.27 ab	23.00 a	11.23 a	HS	HS	S
Om37	96 bc	4.58 ab	33.00 bc	14.29 bc	0	0	MS
L-311(4)	93 ab	4.78 ab	33.20 b	14.19 bc	MS	0	S
LSD5%=	2	1.21	2.76	1.25			

**Table 9:** Values of agronomic parameters and resistance to fungal pathogens in alloplasmic sister DH lines of three families of three hybrid combinations in 2018. Controls: variety Omskaya 33, lines Om37 and L-311(4). (0: immune; R: resistant; MR: moderately resistant; MS: moderately susceptible; S: susceptible; HS: highly susceptible). Different letters within the same column indicate statistically significant differences (*p* < 0.05).

Genotype and Sister DH Lines	Heading Date	Yield (t/ha)	1000-Grain Weight, (g)	Protein Content, (%)	Resistance by Fungal Pathogens		
					Leaf Rust	Stem Rust	Powdery Mildew
**L-311(4) × Om37**							
R17-DH1	89 ab	4.56 a	35.9 ab	15.57 ab	0	R	MR
R17-DH2	90 ab	4.74 a	38.40 b	17.13 b	R	R	MS
R17-DH3	88 a	4.80 a	36.70 ab	15.73 ab	0	R	MR
R17-DH4	92 ab	5.03 a	39.90 bc	15.16 ab	0	0	MR
**L-311(4) × L-134**							
R28-DH1	93 ab	4.66 a	40.70 bc	16.10 b	R	0	0
R28-DH2	95 b	4.80 a	39.70 bc	16.19 b	R	0	0
**L-311(4) × 2870**							
R51-DH1	97 b	5.07 a	39.90 bc	16.90 b	0	0	R
R51-DH2	98 b	4.76 a	37.00 a	16.40‘b	0	0	R
R51-DH3	96 b	4.97 a	39.80 bc	17.56 b	0	0	0
R51-DH4	98 b	5.12 a	39.40 b	17.11 b	0	0	0
**Controls**							
Omskaya33	92 ab	2.78 a	33.10 a	13.67 a	HS	HS	MS
Om37	96 b	3.80 a	34.80 a	15.61 ab	R	R	MS
L-311(4)	94 ab	5.12 a	44.20 c	15.40 ab	MR	0	MS
LSD5%=	3	1.38	2.35	1.14			

**Table 10:** Values of agronomic parameters and resistance to fungal pathogens in alloplasmic sister DH lines of three hybrid combinations in 2019. Controls: variety Omskaya 33, lines Om37 and L-311(4). (0: immune; R: resistant; MR: moderately resistant; MS: moderately susceptible; S: susceptible; HS: highly susceptible). Different letters within the same column indicate statistically significant differences (*p* < 0.05).

Genotype and Sister DH Lines	Heading Date	Yield (t/ha)	1000-Grain Weight, (g)	Protein Content, (%)	Resistance to Fungal Pathogens		
					Leaf Rust	Stem Rust	Powdery Mildew
**L-311(4) × Om37**							
R17-DH1	97 ab	4.51 ab	38.4 b	17.31 b	R	R	MR
R17-DH2	94 a	4.75 ab	38.4 b	15.73 a	0	0	MR
R17-DH3	94 a	4.73 ab	36.1 b	17.10 b	0	0	MR
R17-DH4	94 a	5.02 b	39.9 b	15.16 a	0	0	MR
**L-311(4) × L-134**							
R28-DH1	102 b	4.20 ab	39.4 b	16.70 b	0	0	R
R28-DH2	98 ab	4.55 ab	44.5 c	16.53 ab	0	0	R
**L-311(4) × 2870**							
R51-DH1	90 a	4.72 ab	40.4 b	16.71 b	0	0	0
R51-DH2	91 a	5.08 b	40.3 b	17.61 b	0	0	0
R51-DH3	91 a	4.98 b	39.2 b	17.43 b	0	0	0
R51-DH4	92 a	5.00 b	38.9 b	17.56 b	0	0	0
**Controls**							
Omskaya 33	93 a	3.53 a	24.8 a	14.36 a	HS	HS	S
Om37	96 ab	4.40 ab	37.9 b	15.56 a	0	0	MR
L-311(4)	94 a	4.63 ab	38.4 b	16.02 ab	MS	0	S
LSD5%=	3.0	0.62	2.33	1.10			
